# Role of CTSA institutes and academic medical centers in facilitating preapproval access to investigational agents and devices during the COVID-19 pandemic

**DOI:** 10.1017/cts.2021.15

**Published:** 2021-02-26

**Authors:** Misty Gravelin, Jeanne Wright, M.E. Blair Holbein, Marlene Berro, Jennifer S. Brown, George A. Mashour, Kevin J. Weatherwax

**Affiliations:** 1 Michigan Institute for Clinical & Health Research, University of Michigan, Ann Arbor, MI, USA; 2 Data and Population Sciences, University of Texas Southwestern, Dallas, TX, USA; 3 Clinical and Translational Institute, University of California Los Angeles, Los Angeles, CA, USA; 4 Clinical Research Quality, School of Medicine Research Office, Stanford University, Stanford, CA, USA

**Keywords:** COVID-19, CTSA, academic medical centers, regulatory support, preapproval access

## Abstract

**Introduction:**

With no approved treatments for COVID-19 initially available, the Food and Drug Administration utilized multiple preapproval pathways to provide access to investigational agents and/or medical devices: Expanded Access, Emergency Use Authorizations, and Clinical Trials. Regulatory units within an Academic Medical Center (AMC), including those part of the Clinical and Translational Science Award (CTSA) consortium, have provided support for clinicians in navigating these options prior to the pandemic. As such, they were positioned to be a resource for accessing therapies during the COVID-19 public health emergency.

**Methods:**

A small survey and a follow-on poll of the national Investigational New Drug (IND)/Investigational Device Exemption (IDE) Workgroup were conducted in October and December 2020 to determine whether CTSA regulatory units assisted in facilitating access to COVID-19 therapies and the extent of pandemic-related challenges these units faced.

**Results:**

Fifteen survey and 21 poll responses were received, which provided insights into the demands placed on these regulatory support units due to the pandemic and the changes required to provide critical support during this and future crises. Key changes and lessons learned included the importance of regulatory knowledge to support the institutional response, the critical need for electronic submission capacity for Food and Drug Administration (FDA) documents, and the nimble reallocation of regulatory and legal resources to support patient access to investigational agents and/or medical devices during the pandemic.

**Conclusion:**

AMC- and CTSA-based regulatory units played a meaningful role in the COVID-19 pandemic but further unit modifications are needed for enabling more robust regulatory support in the future.

Key Lessons Learned from the COVID-19 Pandemic

- *Deep Regulatory Knowledge is Critical During a Public Health Crisis*
- *Electronic Submission Capabilities to the FDA are Critically Needed for Efficient Access to Experimental Therapeutics*
- *Nimble Reallocation of Regulatory and Legal Resources Is Necessary to Enhance Patient Access during Periods of High Demand for Investigational Drugs/Devices.*

## Introduction

When COVID-19 appeared in the US in January 2020, clinicians had few therapeutic options to provide treatment for their patients [1]. There were no approved products, no clinical trials, and little information on a novel virus from a family of pathogens where the few strains that infect humans vary dramatically in virulence [2]. Academic medical centers (AMCs) across the country responded with clinical trials and the Food and Drug Administration (FDA) mobilized to deploy every regulatory resource available. Even now, nearly a year into a global pandemic, only one drug (remdesivir) has been approved. Other products, including diagnostics and therapies, are being made available through preapproval mechanisms. The safety and efficacy of these unapproved products remain unknown but each of these preapproval mechanisms require that the potential benefit to the patient outweigh the anticipated risk.

Here we provide an overview of these preapproval mechanisms through the FDA as well as the role of the Clinical and Translational Science Award (CTSA) consortium regulatory support units. We also report the results of a survey and poll that assessed the extent of support these units provided in facilitating access to investigational agents and/or medical devices for COVID-19 and what challenges and changes these units addressed in order to continue to support FDA submissions during the pandemic.

### Preapproval Access to Investigational COVID-19 Agents and Devices

#### Expanded Access

Expanded Access (EA) is a FDA process to provide investigational drugs for treatment use in patients who have a serious condition, who do not qualify for clinical trials, and who have no satisfactory clinical options available. Sometimes called “compassionate use,” this pathway has been available informally since the 1970s but was formalized in 1987 in response to the HIV/AIDS crisis. The modern process was codified in the regulations in 2009 and allows for three types of EA: single-patient (including single-patient emergency use), Intermediate-sized Patient Population, and Treatment Protocols, the latter two more commonly known as Expanded Access Programs [3]. Each type roughly corresponds to the size of the population seeking treatment, whether small, medium, or large. Between 2015 and 2019, the FDA received more than 1000 such requests each year, most of which were to treat individual patients [4]. Devices are also available through EA, although they are not requested as frequently. This includes devices used for direct treatment as well as those intended for diagnosis, such as *in vitro* diagnostic tests. For all therapies provided through EA, regulatory requirements for investigational agents and/or medical devices still apply, including the need for IRB review, written informed consent, and product accountability.

When COVID-19 first affected the US population, EA was the only way to access promising new therapies. Remdesivir was available first through single-patient requests and then through a Gilead Expanded Access Program starting in March 2020 [5]. Convalescent plasma had long been available through single-patient requests for epidemic conditions such as influenza and Ebola [6]. Numerous sites organized Intermediate-sized Patient Population EA for their patients. For example, in early April the Mayo Clinic organized a massive such program, under which tens of thousands of patients were treated at sites across the country [7]. Both of these agents would progress to see widespread use under FDA Emergency Use Authorizations [8,9].

#### Emergency Use Authorizations

The Emergency Use Authorization (EUA) was, prior to the COVID-19 pandemic’s arrival early 2020, a relatively rare type of preapproval access and seldom used at AMCs. An EUA authorizes emergency use of an unapproved medical product, or the unapproved use of an approved medical product, for specific emergency circumstances, following a declaration by the HHS Secretary [10,11]. Use of therapies under EUA do not require the same regulations as either EA or clinical trials, with no IRB review and a simple “fact sheet” provided to the patient in lieu of written informed consent. However, these products are still considered investigational agents and/or medical devices for all other purposes. Since the COVID-19 Medical Countermeasures Declaration (February 4, 2020) and the subsequent Emergency Use Authorization Declaration (March 27, 2020), the FDA has used EUAs for several types of regulated products as a quick and efficient way to make available diagnostic tests, unapproved drugs and biologics, and unapproved devices in order to meet an urgent need. Over 300 *in vitro* diagnostic EUAs (as of January 28, 2021), several dozen types of medical devices including personal protective equipment and ventilators, and a handful of drug and biologic products have been authorized under this mechanism for COVID-19 [8–19]. (Tables 1 and 2) As described above, remdesivir was granted an EUA in May 2020 and eventually received full marketing approval as Veklury® in October 2020 [8,20]. Convalescent plasma was authorized as a therapy for COVID-19 under an EUA in August 2020 [9].

**Table 1.** Emergency use authorizations for COVID-19 (as of January 28, 2021)

Type of EUA	Number	Comments
*In vitro* diagnostic products	323	First issued to the CDC for a PCR diagnostic panel
Personal Protective Equipment and Related Devices	26	NIOSH-approved air purifying respirators for use in health care settings (re-issued twice)
Ventilators and Other Medical Devices	27	Including anesthesia machines and positive pressure breathing devices for use as ventilators
Drug and Biologic Products	10	See Table 2

EUA, Emergency Use Authorization; CDC, Centers for Disease Control and Prevention; PCR, polymerase chain reaction; NIOSH, National Institute for Occupational Safety and Health.

**Table 2.** Select examples of emergency use authorizations (as of January 28, 2021)

Drug/Biologic	Intended Use	Date Issued	Comments
Hydroxychloroquine Sulfate and Chloroquine Phosphate	Treatment of hospitalized patients with COVID-19.	3/28/2020	EUA withdrawn 6/15/2020
Fresenius Medical, multiFiltrate PRO System and MultiBic/MultiPlus Solutions	Continuous renal replacement therapy to treat patients in an acute care environment during the COVID-19 pandemic.	4/30/2020	
Remdesivir for Certain Hospitalized COVID-19 Patients	Emergency use by licensed healthcare providers for the treatment of suspected or laboratory-confirmed COVID-19 in hospitalized pediatric patients weighing 3.5 kg to less than 40 kg or hospitalized pediatric patients less than 12 years of age weighing at least 3.5 kg.	5/1/2020	Reissued 10/22/2020 for the populations for whom Veklury was not approved
Fresenius Kabi Propoven 2%	Maintain sedation via continuous infusion in patients older than age 16 with suspected or confirmed COVID-19 who require mechanical ventilation in an ICU setting.	5/8/2020	
REGIOCIT Replacement Solution that Contains Citrate for Regional Citrate Anticoagulation of the Extracorporeal Circuit	As a replacement solution only in adult patients treated with continuous renal replacement therapy, and for whom regional citrate anticoagulation is appropriate, in a critical care setting.	8/13/2020	
COVID-19 Convalescent Plasma	For the treatment of hospitalized patients with Coronavirus Disease 2019 (COVID-19).	8/23/2020	
Bamlanivimab	For the treatment of mild-to-moderate COVID-19 in adult and pediatric patients with positive results of direct SARS-CoV-2 viral testing who are 12 years of age and older weighing at least 40 kilograms (about 88 pounds), and who are at high risk for progressing to severe COVID-19 and/or hospitalization.	11/9/2020	Manufactured by Eli Lilly
Baricitinib in Combination with Remdesivir	For emergency use by healthcare providers for the treatment of suspected or laboratory-confirmed COVID-19 in hospitalized adults and pediatric patients 2 years of age or older requiring supplemental oxygen, invasive mechanical ventilation, or extracorporeal membrane oxygenation (ECMO).	11/19/2020	Manufactured by Eli Lilly
Casirivimab and Imdevimab	To be administered together for the treatment of mild to moderate coronavirus disease 2019 (COVID-19) in adults and pediatric patients (12 years of age and older weighing at least 40 kg) with positive results of direct SARS-CoV-2 viral testing, and who are at high risk for progressing to severe COVID-19 and/or hospitalization.	11/21/2020	Recombinant human IgG1 monoclonal antibodies manufactured by Regeneron
Pfizer-BioNTech COVID-19 Vaccine	For the prevention of 2019 coronavirus disease (COVID-19) for individuals 16 years of age and older.	12/11/2020	
Moderna COVID-19 Vaccine	For the prevention of Coronavirus Disease 2019 (COVID-19) for individuals 18 years of age and older.	12/18/2020	

EUA, Emergency Use Authorization; ICU, intensive care unit.

#### Clinical Trials – Traditional IND and IDE Applications to the FDA

Investigational New Drug (IND) and Investigational Device Exemption (IDE) applications are requests submitted to the FDA to allow administration of an investigational agent and/or use of a medical device in humans under a research protocol. Clinical trials under IND and IDE approvals are the primary means to collect safety and effectiveness data for the future marketing approval of new drugs, biologics, and devices. They are also required for existing agents and devices that are intended to be used for a new indication, applied to a new population, or through an alternative route of administration and/or with a change in dosage. Data collected under IND and IDE applications can then be used to submit for marketing approval (New Drug Applications, Biologics/Product License Applications, and Premarket Approvals) or EUA.

IND submissions are divided into two categories, research and commercial. The main differences depend on who submits the application to the FDA and the intended purpose of the clinical research. In an AMC setting where the investigator is also the IND sponsor, IND applications are generally considered research as the purpose is typically basic characterization of the safety of new compounds or examining clinical efficacy for a new indication. These are not part of a product development plan and do not focus on data generation for marketing approvals. The “research” IND is usually smaller and less complex than the “commercial” IND application, and electronic submission is not required. By contrast, “commercial” INDs are typically submitted by a drug company or a national sponsor (e.g., NIH or large consortium) and often involve larger scale multi-center clinical trials with complex protocols.

Clinical trials under IND or IDE applications require more extensive protections for subjects than are required for patients receiving treatment under EA or EUA. These protections are necessary because, unlike the other forms of preapproval access, the primary purpose of clinical trials is not to treat patients but to evaluate the therapy, and as such they may have a control arm that receives standard of care therapy or a placebo. At a minimum, these safety measures include IRB review and approval, written informed consent, monitoring for compliance with regulations and patient safety, and the maintenance of equipoise in treatment between therapeutic groups.

### Regulatory Resources at CTSA Consortium Hubs

The NIH established the Clinical and Translational Science Award (CTSA) program to create a nationwide consortium intended to accelerate the translation of scientific discoveries to improved health. Essential to this effort were collaborative work groups established to address identified barriers in clinical and translational research. One such workgroup was charged with addressing the unique challenges of research using FDA-regulated agents by investigators in AMCs. The IND/IDE Taskforce, within the now discontinued Regulatory Knowledge and Support Key Function Committee, was assembled with regulatory specialists from the individual CTSA hubs [21]. The CTSA network has grown from 12 original hubs to the current 62, and among the CTSA hubs, the level of support for IND/IDE applications varies widely [22]. A 2008 survey of 24 existing hubs with 19 respondents indicated that many hubs provided some level of support with a small number providing comprehensive support [21].

As the CTSA network matured, the formal IND/IDE Taskforce transitioned into an informal IND/IDE Workgroup. This legacy workgroup has continued to regularly meet, provide educational sessions, and develop and disseminate IND/IDE best practices with active participants from the majority of current CTSA hubs as well as other AMCs. The support level provided to study teams by these regulatory staff continues to vary across institutions. A recent survey of the IND/IDE Workgroup asking about local resources to support EA applications also revealed a wide range of resources at an institutional level, ranging from minimal to full support, including completion and submission to the FDA and IRB for these requests (unpublished data).

In addition to regulatory expertise provided at individual hubs, the National Center for Advancing Translational Sciences funded a U01 Collaboration and Innovation award called TEAMSS (Transforming Expanded Access to Maximize Support and Study) [23], the focus of which is the development and national dissemination of best practices for creating preapproval access to investigational agents and medical devices.

## Methods

To assess the response of CTSA regulatory support units to the COVID-19 crisis, a survey was prepared in Qualtrics with ethics approval (University of Michigan IRBMED, HUM00189448). Questions focused on the experience of regulatory staff in responding to the COVID-19 pandemic, including number and types of submissions related to COVID-19 treatment, new or altered workflows, as well as a qualitative assessment of the benefits of these changes for this and future circumstances. The survey was sent via email to regulatory staff at AMCs that participate in the IND/IDE Workgroup, primarily centered around the CTSA network Regulatory Knowledge and Support cores. Responses were accepted between October 19 and October 30, 2020.

Descriptive statistics were performed on quantitative responses, and qualitative responses were coded for themes. These responses were then used to construct multiple-choice questions based on the responses to the survey, which were presented as poll questions to the participants at the December 14, 2020 IND/IDE Workgroup meeting.

## Results

### COVID-19 Response at CTSA and Academic Medical Center Regulatory Support Units

Fifteen responses to the survey were received, representing 13 distinct institutions, of which 10 are CTSA hubs. This represents 16% response rate for CTSA hubs (10/62). The poll questions were distributed anonymously to the 38 participants at the December IND/IDE Workgroup meeting, of which 21 responded to the majority of questions.

Among the survey respondents, all (n = 15) had existing site infrastructure for traditional research regulatory submissions (IND and IDE) and EA. Two-thirds (n = 10/15) reported existing infrastructure to support Emergency Use Authorizations as well. A clarifying question was presented to the poll participants, who reported that only 3 of 18 had actually submitted an EUA prior to the pandemic.

Most survey respondents endorsed workflows being developed or expanded in response to the pandemic (n = 11; 73%), with other processes streamlined (n = 8; 83%), but only two (13%) reported entirely new resources. Among the qualitative reports, 5 of 15 specifically reported prioritization of protocols for COVID-19 treatment, whether directly through the regulatory support or through other institutional committees. When these were presented to the poll respondents, most reported similarly expanded workflows (n = 12; 57%) and formal or informal prioritization (n = 12; 57% for both). However, electronic submission and remote monitoring, which were previously mentioned in qualitative survey comments, were the most endorsed changes in the poll (respectively n = 13 and 14; 62% and 67%). Survey and poll responses are compared in Table 3. Notably, during the poll, the additional concern over lost resources or lost staff due to economic recovery from the pandemic was expressed and spontaneously endorsed by three other participants.

**Table 3.** Changes in response to COVID-19

	Survey (n = 13)		Poll (n = 21)	
Theme	Number	Percent	Number	Percent
Practices developed or expanded*	11	85%	12	57%
Workflows changed or streamlined*	8	62%	9	43%
New resources added*	2	15%	5	24%
Mandatory requirements for submission added*	2	15%	3	14%
Informal prioritization of COVID projects/protocols	2	15%	12	57%
Formal prioritization of COVID projects/protocols	2	15%	12	57%
Remote monitoring implemented	1	8%	14	67%
Electronic submission implemented or expanded	1	8%	13	62%
Mandatory requirements for submission removed*	0	0%	1	5%
No changes were made*	3	23%	0	0%

* Available as fixed selection in survey.

Key lessons learned from this experience were more aligned between the free-text survey and the poll responses. The most commonly reported lesson in both was the necessity of electronic submission of documents to the FDA, either through the NextGen Portal or the Electronic Submission Gateway, which was also mentioned by one additional respondent as a valuable change in practice in the previous question. The need for general flexibility during this time was also highly endorsed as well as the critical importance of existing regulatory knowledge or expertise. Themes from the survey responses and poll answers are listed in Table 4.

**Table 4.** Themes among key lessons learned

	Survey (n = 13)		Poll (n = 21)	
Theme	Number	Percent	Number	Percent
Need for electronic submission	4	31%	15	71%
Flexibility	3	23%	10	48%
Importance of prioritization	2	15%	4	19%
Lack of resources for the required level of response	2	15%	9	43%
Importance of regulatory expertise	2	15%	14	67%
Need for more institutional guidance	1	8%	5	24%
Need for more FDA guidance	1	8%	6	29%

FDA, Food and Drug Administration.

Survey participants were also asked which changes were specifically problematic and should be discontinued. Five responded that none of the changes should be discontinued. Three addressed the need for more systematic processes in the future, including one specifically referencing the missed opportunity for prioritization as mentioned in the key lessons learned. Similarly, two more expanded on the experience of learning that the new processes were not sustainable in the long term. This question was not presented to the poll participants.

The majority of respondents in both the survey and poll indicated that they would be prepared for FDA submissions to respond to a future crisis (Survey: n = 12; 92%. Poll: n = 15/18; 83%). Only the survey respondents were asked for recommendations for future public health emergencies, but their answers echoed the lessons that had been learned in changes already implemented: flexibility (n = 2; 17%), prioritization (n = 3; 25%), and adjustments in resource allocation to ensure a sustainable response (n = 3; 25%). The most common recommendation was the development of policy and the need for pre-planning, at both the institutional and agency level (n = 4; 33%).

The final block of questions asked specifically about experience with certain regulatory submissions for treatment of COVID-19. Most respondents in both the survey and poll had submitted INDs for investigator-initiated studies and single-patient requests for expanded access. Fewer had participated in an EUA. Table 5 shows the number of responses for each submission type. When asked for the number of submissions that the site had supported, the number varied widely for each category (Table 6). Poll respondents were not asked this question. All respondents, regardless of experience with submissions, had been asked to provide their expertise on regulatory pathways to assist study teams developing and testing treatments.

**Table 5.** Experience with different regulatory submissions for COVID-19 Treatments

	Survey (n = 13)		Poll (n = 18)	
Type of Submission	Had Submitted	Percent	Had Submitted	Percent
IND for investigator-initiated studies	11	85%	16	89%
IDE for investigator-initiated studies	1	8%	5	28%
Emergency Use Authorization	8	62%	6	33%
Single-patient Expanded Access	11	85%	16	89%
Multi-patient Expanded Access	2	15%	6	33%

IND, Investigational New Drug; IDE, Investigational Device Exemption.

**Table 6.** Number of regulatory submissions for COVID-19 treatments (n = 13)

Type of Submission	Median	Range
IND for investigator-initiated studies	3	1–10
IDE for Investigator-initiated studies	3	3
Emergency Use Authorization	2	2–6
Single-patient Expanded Access	6	2–50
Multi-patient Expanded Access	2.5	1–5

IND, Investigational New Drug; IDE, Investigational Device Exemption.

Specific to remdesivir, respondents reported participating in all stages of preapproval access: single-patient expanded access (n = 8; 62%), participation in the multi-site expanded access program (n = 9; 69%), and EUA responsibilities (n = 6; 46%). Two sites submitted single-patient requests but did not participate in the multi-patient program, while three sites participated in the multi-patient program but did not submit any single-patient requests. Four sites reported participation in all three stages. Poll respondents were not asked about these programs specifically.

## Discussion

The current COVID-19 pandemic highlighted the complexities and multiple pathways for use of non-FDA-approved drugs and devices. There was an immediate need to treat desperately ill patients but treatment options often carried significant regulatory requirements and potential delays. These concerns were stacked on the complexity of the regulatory environment, including the range of COVID-19 FDA submission types in EA, EUAs, and traditional IND/IDEs. This was compounded by constantly changing guidelines as the FDA sought to increase the availability of needed therapeutic tools, to account for the exponential growth in knowledge of the disease and to speed access to early clinical successes. As a result, regulatory expertise was needed to advise both the clinical and research environments in the use of investigational agents and/or medical devices.

### Key Lesson Learned: The Importance of Deep Regulatory Knowledge

Institutions with a CTSA hub often turned to them for regulatory support to address these challenges. The longstanding expertise of regulatory support units was suddenly critical to many aspects of the pandemic response, as reported by the survey and poll respondents, often in ways that were novel for the institution. The most obvious example of this is the need for detailed understanding of EUAs, which serve an expressly clinical purpose. However, the clinical enterprise at AMCs has no reason to invest in either the foundational or specific knowledge of this mechanism, due to its rarity. CTSA regulatory support units, by contrast, may not have had previous experience but have spent years or decades developing the foundational regulatory expertise, which meant they could rapidly delve into the emerging guidance and develop the needed understanding to advise on the use of investigational agents and/or medical devices in these unique circumstances.

### Key Lesson Learned: Electronic Submission to the FDA

A significant development that was noted positively across responses was the shift to electronic submissions to the FDA. Prior to COVID, the majority of academic investigators submitted their IND applications in paper format, as the primary electronic submission format required expensive software and use of the complex e-CTD (electronic common technical document) format. While the FDA had recently made available alternative electronic submission methods, such as additional formats supported by the Electronic Submission Gateway and the more dedicated CDER NextGen Collaboration Portal, it was the COVID-19 pandemic that forced their rapid uptake throughout the AMCs. With most regulatory staff working from home, the printing and mailing of submissions became untenable and collaborative groups in the CTSA consortium, such as the IND/IDE Workgroup, came together to train one another on these electronic submission processes.

### Key Lesson Learned: Resource Considerations

The survey responses described many dimensions of the sudden centrality of regulatory support as the COVID-19 response became paramount, all of which required additional effort. This included both the increase in services provided as well as the work to support new partnerships with other institutional units such as the IRB, legal teams, and clinical teams. Accordingly, many key changes repeated by survey respondents addressed this overload with ways to streamline submission and support, many of which may be continued even in non-pandemic times, such as enhanced feasibility review, prioritization of studies, and increased coordination between clinical operations and research.

## Limitations

The survey and poll suffer some limitations that may affect the generalizability of these conclusions. From the outset, the survey was sent to the IND/IDE Workgroup, which represents most but not all CTSA hubs. There may be inherent differences between the hubs that participate in this group and those that do not. The survey also had a poor response rate, possibly due to the ongoing demands of the COVID-19 pandemic itself, which may introduce further reporting bias. The poll was developed to address this concern in part, but limitations of the format meant that questions had to be revised and anonymous respondents may or may not have overlapped with survey respondents. As such, survey and poll responses may not always be directly comparable.

## Conclusion

At the time of a public health emergency such as COVID-19, the FDA requires many tools to make agents and medical devices available quickly but this has the potential to complicate the regulatory environment. CTSA regulatory support units have the potential to serve as a critical resource to clinicians as they navigate the pathways to provide access to needed therapies for their patients.
